# Six principles to enhance health workforce flexibility

**DOI:** 10.1186/1478-4491-13-9

**Published:** 2015-04-07

**Authors:** Susan A Nancarrow

**Affiliations:** School of Health and Human Sciences, Southern Cross University, East Lismore, NSW 2480 Australia

**Keywords:** Interdisciplinary, Accessibility, Delegation, Models of care, Therapeutic partitions, Incremental competencies, Micro-specialisation, Professional brand, Portfolio competencies

## Abstract

**Abstract:**

This paper proposes approaches to break down the boundaries that reduce the ability of the health workforce to respond to population needs, or workforce flexibility.

Accessible health services require sufficient numbers and types of skilled workers to meet population needs. However, there are several reasons that the health workforce cannot or does not meet population needs. These primarily stem from workforce shortages. However, the health workforce can also be prevented from responding appropriately and efficiently because of restrictions imposed by professional boundaries, funding models or therapeutic partitions. These boundaries limit the ability of practitioners to effectively diagnose and treat patients by restricting access to specific skills, technologies and services. In some cases, these boundaries not only reduce workforce flexibility, but they introduce inefficiencies in the form of additional clinical transactions and costs, further detracting from workforce responsiveness.

Several new models of care are being developed to enhance workforce flexibility by enabling existing staff to work to their full scope of practice, extend their roles or by introducing new workers. Expanding on these concepts, this theoretical paper proposes six principles that have the potential to enhance health workforce flexibility, specifically:

1. Measure health system performance from the perspective of the patient.

2. Minimise training times.

3. Regulate tasks (competencies), not professions.

4. Match rewards and indemnity to the levels of skill and risk required to perform a particular task, not professional title.

5. Ensure that practitioners have all the skills they need to perform the tasks required to work in the environment in which they work

6. Enable practitioners to work to their full scope of practice delegate tasks where required

These proposed principles will challenge some of the existing social norms around health-care delivery; however, many of these principles are already being applied, albeit on a small scale. This paper discusses the implications of these reforms.

**Proposed discussion points:**

1. Is person-centred care at odds with professional monopolies?

2. Should the state regulate professions and, by doing so, protect professional monopolies or, instead, regulate tasks or competencies?

3. Can health-care efficiency be enhanced by reducing the number of clinical transactions required to meet patient needs?

## Background

The workforce accounts for the greatest proportion of recurrent health expenditure in most health settings. Increasingly, health workforce shortages are corresponding with growing demands on the health-care system and continuing growth in health-care expenditure [1]. Staff shortages not only limit health-care accessibility but are a key factor in health-care quality and safety [2, 3]. Growing health-care costs mean that simply employing and training more of the same types of worker is not a sustainable solution. Instead, innovative solutions are required to use existing workforce resources more efficiently.

Health workforce flexibilities have been introduced variously as a way to increase organisational efficiency, address workforce shortages and better meet patient needs [4]. A flexible workforce is described as one with the ability to ‘employ and pay at normal rates just sufficient labour to meet the workload at any point in time’ [5]. From a patient perspective, truly accessible care relies on the provision of appropriate health care in the right place at the right time [6]. However, the health workforce is relatively inelastic because of the way that tasks and skills are bound to traditional professional role boundaries [7]. This paper explores mechanisms to enhance flexibility within the health workforce.

A truly flexible workforce has the potential to optimise health-care accessibility by ensuring timely responses to labour shortages, by avoiding the lengthy and expensive training regimes required to fully train many health providers and by distributing health resources in a way that more efficiently meets the needs of the patient. The interactions and interrelationships that influence workforce flexibility mean that it is a complex area to examine. For the purpose of consistency, the health workforce referred to in this paper includes any qualified or unqualified person involved in the paid delivery of health or social care, and they are referred to as a ‘practitioner’. This includes the clinical and administrative workforces within allied health, medicine, nursing and social care.

Workforce flexibility is receiving increasing international attention [8]. For instance, rural health workforce shortages have resulted in proposals to remove legal and professional barriers to practice to promote flexible service delivery [9, 10]. A number of health policies explicitly acknowledge the importance of workforce flexibility both to help address workforce shortages and to enhance patient-centred care [11–13]. However, most examples of workforce change tend to involve localised, small-scale projects that take place within the normative frameworks of the existing professional paradigms. They largely involve role negotiation between a small number of professions [7], rather than wholesale redesign of the workforce. There is limited research into systemic approaches to implementing large-scale workforce flexibilities [14].

Some of the most powerful levers of health workforce flexibilities have been unintended. In particular, there are several examples of change initiated by workforce shortages in certain professions resulting from, for example, outbreaks of disease [15, 16], war [17] and major policy changes, such as the European Working Time Directive [18]. The implications of these shortages were the redistribution of roles to other types of workers, growth of new workers and ultimately an expanded repertoire of skills for some [7, 19]. The European Working Time Directive, which placed a cap on the maximum number of working hours for doctors, coincided with several workforce re-engineering projects [20–23].

Other unintended levers of workforce change have included the introduction of contestability in health-care delivery [24, 25]. For example, Borthwick highlights the way that the introduction of new managerialism in the National Health Service (NHS), in the form of general practitioner fundholding, enabled podiatric surgeons to compete with orthopaedic surgeons to secure formal recognition to practice autonomously in the NHS. Podiatric surgeons provided evidence of cost-effectiveness and quality of their services, demonstrating that they were able to help address a range of quality standards including waiting times and patient satisfaction. This was in the face of opposition from the British Medical Association surgeons, who proposed that surgical services should only be provided under the control of medical practitioners.

Other levers for change have included crises in health-care delivery, such as the Bristol Royal Infirmary case of systemic failures which led to the unnecessary deaths of several children undergoing heart surgery [26]. The outcome of this inquiry challenged the way that professions were regulated, and led to new external accountabilities, with a fundamental shift from profession-centric to patient-focused regulation [27].

### Occupational monopolies and occupational imperialism

Common sense, and a growing evidence base, suggests that with appropriate training and supervision, a great deal of health-care can be delivered by more than one type of practitioner [28]. Logic should dictate that any person credentialed to perform a specific task or role should be able to undertake that role. However, the normative model of the health workforce assumes that there is a ‘right’ type of practitioner to deliver specific types of care [29].

The health workforce is organised around groups of professions that embrace a repertoire of pre-defined skills within a unifying philosophy, codified through formal education and measured by the achievement of specific competencies. This principle, known as social closure, is the way that professions create a unique identity and monopolise a body of knowledge [30]. Professions engage in strategies to prevent competitors from encroaching into their task and knowledge domains, while at the same time attempting to adopt skills and roles from competing professions [29, 31, 32]. Consequently, professional repertoires have evolved, historically, through market opportunism and negotiation rather than necessarily on the basis of logic, evidence or patient needs [7, 33]. Social closure is upheld by state acceptance and endorsement of professions through legislation and regulation [34]. In other words, the professions become a type of state-endorsed brand, where a *professional brand* is seen as a unique marketing message that conveys a sense of perceived value for consumers and differentiates it from other providers of those products.

One consequence of the monopoly afforded by social closure is an underlying assumption that those professions that have adopted a specific repertoire of skills are the *only* group of practitioners with the ability or authority to perform those tasks. This is illustrated by the demand for evidence that a credentialed worker can safely and effectively perform tasks that have formed the traditional skill base of other professions. For example, over the past decade, a great deal of research and published literature has been dedicated to demonstrating that nurses can perform tasks as effectively as doctors [35]. There are also several illustrations of role renegotiation and turf wars between doctors, allied health, nursing and the assistant workforce [36], reinforcing the assumption of ‘ownership’ of specific core tasks by particular workers. These socially constructed role boundaries reduce workforce flexibility by limiting task and role transfer between different types of practitioners. To help overcome this, workforce planning tools have been developed that deconstruct tasks and reallocate them to workers based on levels of risk and patient need, rather than traditional role boundaries [37, 38]. In many cases, the renegotiation of roles results in professions retaining tasks that they see as being core to their professional identity while discarding those roles that they see as dispensable [25, 31, 39]. The relative ability of different professions to negotiate and maintain their ‘brand’ depends largely on their level of state protection and the degree of influence of one profession over another [29, 40–42].

### Barriers to flexibility

Patient care can be compromised when health practitioners lack access to the full range of skills or technology necessary to fully achieve their therapeutic goals [26]. Instead, they have to refer to a suite of other practitioners to achieve their therapeutic or treatment goal, referred to here as a *therapeutic partition*. In health, therapeutic partitions arise where practitioners lack access to appropriate diagnostic investigations (such as diagnostic radiography or pathology), are prevented from performing minor procedures (such as suturing), are restricted from accessing medication that is needed to fully treat a patient or are unable to make direct referrals to other practitioners or institutions [43]. The consequence of therapeutic partitions is that patients have to make multiple clinical transactions to achieve a single therapeutic goal. Therapeutic partitions risk being more expensive [44] and more time-consuming than having a diagnosis and intervention provided by a single appropriately skilled practitioner or co-located team. There has been little explicit examination of therapeutic partitions; however, the growth of approaches designed to streamline and integrate health services is an implicit acknowledgement of this problem. In settings that normally involve multiple care transitions, or ‘handovers’, such as in emergency care, it is well acknowledged that these points create vulnerabilities in care delivery [28].

Some of the causes of therapeutic partitions are regulatory, such as restricted access to prescribing. Health financing models are also responsible for built-in inefficiencies. Health services that are funded variously by different agencies, such as federal–state or public–private payers, create perverse incentives for organisations to shift costs between sectors and providers, which are not necessarily in the best interests of the patient [29, 45]. Therapeutic partitions can result from funding models that limit state reimbursement for services to referrals made by specific types of practitioners. For example, state reimbursement for radiographs may be limited to referrals by medical practitioners, even though others, such as physiotherapists or podiatrists, may be credentialed to order these diagnostic tests [43]. Similarly, general practitioners (GPs) have been restricted from directly accessing magnetic resonance imaging (MRI) in some jurisdictions, yet evidence shows that patients whose GPs could access MRI achieved slightly better outcomes [46] and care was more cost effective [44]. Consequently, a more expensive service (such as a medical practitioner or medical consultant) becomes an additional clinical transaction in the achievement of the diagnostic goal.

More expensive or specialised services are often explicitly or implicitly rationed by requiring patients to obtain referrals to access these services. However, the referral itself creates an additional clinical transaction with a financial and opportunity cost for the patient and health system. This is particularly inefficient when referrals are required to access practitioners who are otherwise ‘first-contact’ practitioners. For instance, in Australia, direct state reimbursement for many allied health services is only possible following assessment and referral by a GP, after completing a client care plan [30]. There are several examples of otherwise qualified practitioners being unable to make direct referrals to other practitioners and services, such as hospital admissions [47].

However, in many cases, there are no regulatory barriers to new or more efficient ways of working. For instance, non-medical prescribing is available in several jurisdictions with some additional training and credentialing [31, 32]; and many allied health professionals can directly order diagnostic radiographs; Instead, the inefficiencies arise from historic ways of working, interdisciplinary role disputes, by more dominant health professions exerting control over the roles of other workers [12, 35, 36], the provision of state funding for some services and not others, the failure to fully delegate work to a lower cost worker when appropriate [39] and inefficient therapeutic partitions.

There have been some attempts to overcome these inefficiencies by better coordinating care [29] and creating integrated service models [37]. Interdisciplinary teamwork can facilitate the blurring of role boundaries, and better understanding of other workers’ roles, so that team members can more flexibly meet patient needs [48, 49]. However, the complex renegotiation of roles through interdisciplinary engineering and retrofitted models of service integration could be seen as a system failure in instances when, instead, it may be possible for a single, appropriately trained practitioner or an appropriately organised team to do the job.

### Models of labour flexibility

Atkinson’s model of labour market flexibility is widely used and describes five domains of workforce flexibility [50]:

1. *External numerical flexibility*, which refers to adjustments to the overall workforce capacity. In the health workforce, this typically takes place through modifications to training and facilitating workforce migration. The European Working Time Directive was one initiative which had a major flow-on effect to numerical flexibility across the health workforce [18].
2. *Internal numerical flexibility*, which involves structural adjustments to the workplace that allow workforce capacity to adjust to service needs. For instance, flexibility in team structures enable staff to be deployed to the areas of greatest need at the appropriate time and reduce the amount of lost activity due to downtime. Examples include the introduction of flexible terms and conditions of employment, such as annualised hours, employee self-rostering and strategies to reduce staff turnover.
3. *Functional flexibility* is the extent to which staff roles are transferrable within an organisation. Delegation and role substitution is used increasingly as a mechanism to increase the flexibility of the health workforce [7]. However, functional flexibility is often limited by therapeutic partitions arising from regulatory and financial boundaries around the nature of health work.
4. *Locational flexibility* examines flexibility in terms of the location of working and is becoming increasingly important in the delivery of accessible health services to people with geographic or other restrictions. Telehealth is becoming an important intervention to address locational flexibility [51].
5. *Wage flexibility* is the determination of pay levels based on demand and the individual costs of employment, rather than on a collective basis.

The changing nature of employment contracts is another way to introduce workforce flexibility [52]. Examples of contractual approaches to flexibility include ‘outsourcing’ the workforce, where the employer sub-contracts the provision of labour to an intermediary such as an agency. Specific flexibilities can be built into employer–employee contracts to accommodate fluctuations in service demand. The risk of these flexible models is that service flexibility is provided at the expense of employment conditions for workers. Additionally, changing contractual accountabilities between workers and employers introduces new requirements for monitoring the quality and outcomes of care [53].

These domains explain several of the levers that can be used to influence health workforce flexibility as part of a concept known as the ‘flexible firm’ in which workforce flexibility is used to drive cost containment and demand management [4]. The flexible firm enables employers to achieve flexibility [54] through segmenting the workforce into a ‘core’ of full-time employees with a ‘periphery’ of part-time or sub-contracted workers [55]. However, this model has been criticised on the basis that the core–periphery dualism belies the complexity of workforce restructure [55] and that ‘peripheral’ flexibilities are being applied to a wider cadre of occupational categories than initially assumed [56].

This potential for workforce flexibility is reflected in the growth of the delegatory workforce, such as support workers, including therapy assistants, health-care assistants and therapy aides [57]. It is reinforced through competency-based frameworks such as the Calderdale Framework, which triages worker skills to meet patient needs based on skills and risk, not traditional role boundaries [37].

In addition to introducing new roles, there is mounting evidence that more efficient use of staff can benefit the patient and the health service. For example, involving extended scope GPs [58] and allied health practitioners in the triage, assessment and treatment of patients on specialist/surgical waiting lists can reduce patient waiting times and result in more appropriate referrals to medical specialists [59]. There are other examples of practical role shifting to enhance health-care efficiency. For instance, a growing number of non-radiographers perform diagnostic ultrasound [60], pharmacists can prescribe in some jurisdictions [61] and foot surgery is performed by some podiatrists [24].

Workforce shortages, alongside an increasing focus on patient-centred care, call for a labour supply that can respond to patient needs, rather than just practitioner, service or organisational drivers. Increasingly, traditional health-care providers are allocating tasks to other practitioners, such as support workers, freeing the more highly trained practitioners to manage patients with complex needs. However, health workforce responsiveness is hampered by historic models of role delineation which are strongly socially entrenched and reinforced by regulatory and funding models.

## Six principles to enhance workforce flexibility

To address the points raised above, this paper proposes six interrelated principles that can be considered to promote role flexibility in the health workforce. The six principles described here differ from the traditional approaches to achieving flexibility because they start from the perspective of the patient, specifically:

1. Measure health system performance from the perspective of the patient.
2. Minimise training times.
3. Regulate tasks (competencies), not professions.
4. Match rewards and indemnity to the levels of skill and risk required to perform a particular task, not professional title.
5. Ensure that practitioners have all the skills they need to perform the tasks required to work in the environment in which they work.
6. Enable practitioners to work to their full scope of practice delegate tasks where required.

### Principle 1: Measure health system performance from the perspective of the patient

There have been widespread moves to implement patient-centred approaches to care and move away from traditional, institutionally based, hierarchically focused models of health-care delivery. However, inefficiencies arise because of traditional role boundaries that result in patients being passed between a range of different professionals to achieve a therapeutic end [14].

Several recent examples of minor workforce innovations illustrate how a small shift in emphasis from the practitioner to the patient can facilitate improvements. A good example is telehealth, which is widely credited with reducing travel times and travel costs for patients [51]. However, as the costs of travel are largely borne by the patients, not the services, these benefits are rarely factored into decisions to implement telehealth if the efficiencies are not to be translated into reduced service costs [62]. Similarly, the effectiveness of workforce reform is often difficult to capture because of the downstream effects of changing health service processes [14]. For example, there is evidence that using allied health professionals to triage surgical waiting lists can reduce patient waiting times; at the same time, such services increase the likelihood that patients referred to the surgeon will require surgery [59]. The system level result of this approach is a condensation of demand for surgical services because a higher proportion of the surgeon’s workload requires more intensive intervention. This in turn increases the cost of those services because more of the surgeon’s time is spent delivering more expensive services. However, the societal benefits of reducing the rates of disability that may arise from having large numbers of patients untreated on a waiting list for a long period are rarely measured or factored into decisions around waiting list management [14].

Consistent approaches are required to measure and capture the outcomes of workforce change interventions [14]. Focusing on the needs of the end user, rather than the service or practitioner, will promote the development of measures to standardise the way that workforce change interventions are described and enable comparison between interventions.

Unless the entire health system starts from the principle of ensuring that the outcome is optimal for the patient, widespread reform and efficiencies are difficult to achieve. While a great deal of rhetoric surrounds the ideals of patient-centred care, the funding models and fragmentation of health-care in many health systems do little to support this principle.

The implementation of this principle is likely to rely on an unambiguous, macro (policy) level focus on meaningful, patient-centred goals of health-care delivery, such as accessibility (including waiting times and care close to home) and quality. For example, various international policy targets to reduce waiting times for elective surgery and in emergency care have resulted in system changes that have successfully reduced waiting times for patients [63–65], several using workforce flexibilities. Performance targets based on quality and accessibility in primary health care in the United Kingdom NHS led to improvements in some aspects of care, in part, through increased workforce flexibility, although the benefits of these were not sustained under a pay-for-performance scheme [66]. Regulatory systems should uphold this principle by privileging patient safety above professional protection.

The shift towards truly patient-centred measures of health system effectiveness still has some way to go. However, the relatively recent introduction of funding models that follow the patient, rather than the system, may enhance patient-centred models of care.

### Principle 2: Minimise training times

Training is the traditional way to increase external numerical flexibility. Despite widespread workforce shortages, there has been little debate around the relationship between the length or models of health-care training and health workforce flexibility. Long training times increase costs and reduce the responsiveness of the workforce to meet changing needs [67]. Most traditional models of health profession training require students to complete an extended period (normally several years) of university education before they achieve a single, marketable skill [67, 68]. Paradoxically, training times for many health professions have increased in the face of widespread workforce shortages. For instance, many allied health and nursing training programmes in Australia and the USA have expanded from 3- to 4-year undergraduate (bachelor’s degree) training programmes, graduate entry master’s degrees or, in some case, doctoral-level training [68–70]. Similar trends are evident in nursing [71]. In Australia and the UK, post-graduate training for medical practitioners is an increasingly dominant model [22, 23] and is the norm in the USA.

Most health practitioner education requires access to several hundred hours of supervised clinical training. Clinical training infrastructure can be expensive for students [72], training institutions and supervisors. There is evidence that poorly conceived clinical placements are a burden on health service delivery [73], although well-constructed placements may enhance service efficiency [74]. Thus, the models of clinical training also have the potential to enhance or detract from service delivery and capacity.

There is an urgent need to rethink the way that health professionals are trained so that the workforce can respond to needs in a cost-effective and timely way. One innovative solution involves a shift away from the time-based achievement of qualifications to the *incremental* achievement of specific competencies [75]. Flexible training models, such as *step-on*, *step-off* programmes, allow the incremental credentialing of practitioners by providing several exit points throughout the course of professional training. This means that workers have a marketable skill, more quickly, that can be responsive to the context in which they work [76, 77].

Incremental training also facilitates opportunities for *micro-specialisms.* Micro-specialisms involve a short training time to learn a particular task to meet a specific, focused (and often, high volume) need. Examples of micro-specialisms include newborn hearing screening specialists, phlebotomists, cast technicians and foot care assistants.

Incremental training approaches have the potential to increase workforce flexibility by enabling practitioners to take on a repertoire of skills that are appropriate for the context in which they work, rather than dictated by historic professional boundaries. For example, providers of home-based rehabilitation services are often required to deliver tasks that are the traditional domain of another profession [78]. It may make more sense for a single, multi-skilled practitioner to deliver these tasks than bring a team of practitioners to deliver specialised components of the job. These tasks are often negotiated between the professionals and may require additional training. Incremental credentialing enables the practitioner to formally adopt these tasks as a part of a repertoire of skills that is fit for purpose and context.

A recent Australian project to support the introduction of a rural allied health generalist used a structured workforce change tool (the Calderdale Framework) to identify tasks that had the potential to be shared between rural and remote allied health practitioners from six discrete allied health disciplines [39]. The study identified 337 discrete clinical tasks. Of these, 45% were already delivered by more than one profession and 127 tasks were deemed as appropriate for skill sharing across two or more professions. Interestingly, in contrast with the sociological literature that emphasises jurisdictional ‘turf wars’ [7], the tasks were negotiated consensually by the participating professions, which may be associated with workforce shortages due to the rural context and the lack of dominance of any one professional group. The shared tasks were repackaged into 13 categories based on functional and diagnostic categories, rather than traditional professional repertoires. A recommendation of the study was that the 13 reconfigured bundles of tasks should be made available to allied health practitioners to support their more generic roles in rural and remote clinical practice. Incremental credentialing of allied health professionals would support this approach.

The expanding knowledge economy is beginning to challenge the traditional role of higher education in the delivery of professional training [79]. In particular, there is pressure to increase the explicit relationships between education, knowledge production and translation, and economic benefit. Decades ago, Gibbons and colleagues [80] proposed a shift in knowledge production from a unidisciplinary, institutionally, hierarchically based approach (Mode 1) to transdisciplinary, non-hierarchical, reflexive approaches that are based around collaborating to address problems in a specific and localised context (Mode 2). Traditional models of health practitioner training are largely embedded in Mode 1 approaches, focusing on the ownership of a professionally discrete body of knowledge. Interprofessional training approaches suggest a move towards Mode 2 training; however, many of these approaches appear to start from the perspective of professional role renegotiation than from patient need [14].

To help address these issues in the health context, health practitioner training could be shifted from university-based education to predominantly clinically based education, where the trainees contribute to health service delivery while learning their profession with the support of ‘outsourced’ formal university training [77]. At the same time, workforce flexibilities could potentially be increased by shortening training times through the achievement of specific milestones rather than time-based achievement of competencies [75].

### Principle 3: Regulate tasks (competencies), not professions

State regulation of professions, rather than specific competencies, reduces flexibility in two ways. First, professions need to demonstrate competence in a large number of domains before they are deemed fit for practice, prolonging training times. Yet, the achievement of some competencies, incrementally, may provide individuals with skills that are marketable well before they achieve their professional status (such as micro-specialisms). Second, when the bundled competencies become part of a professional repertoire, role boundaries are generally dictated by professional politics and history rather than patient needs [7]. State regulation of professions provides a stamp of legitimacy over the jurisdictional claims of the professions, reinforcing their professional branding.

Several authors have debated the relationship between professional monopolies and state regulation [81–83]. However, few challenge the repertoires that go together to make up the professional portfolio. This principle challenges the assumption that a single profession should have unique ownership over a particular professional repertoire. Instead, the tasks and skills that form that repertoire should be examined and credentialed individually and anyone deemed competent to perform a particular task should be enabled to do so.

The recognition of specific competencies allows for a responsive, flexible workforce in several ways. For example, existing professions can develop a portfolio of competencies that are relevant to their field or practice context. The example of the rural health generalist in Australia is a clear illustration of a portfolio of competencies that cross traditional professional jurisdictions to meet local needs [39].

Disentangling specific competencies from traditional professional repertoires is likely to emphasise the levels of expertise required to perform these roles. This principle does not suggest that highly skilled, high-risk tasks should be available to all practitioners. Indeed, high-risk and expensive tasks would be likely to come under *greater* scrutiny if they were contested on the basis of quality, efficacy and cost, not professional titles (as illustrated by the case of the podiatric surgeons and natural therapies below). Specific tasks could then be clearly governed, monitored and reimbursed around a current evidence base [84].

Similarly, there have been proposals to remove state reimbursement for tasks that are *not* deemed to be evidence based, such as homeopathy [85]. However, the coupling of professions with tasks was reinforced in the scope of a recent Australian review on private health insurance rebates for natural therapies which stated that it included ‘only natural therapies that are *not* provided by a health professional accredited under the Australian Health Practitioner Regulation Agency’ [86]. This explicit coupling of professions with tasks belies the fact that registered professionals often perform tasks that are outside their accepted repertoire [87] and deliver tasks that have a limited evidence base including the use of natural therapies [85]. It risks creating inequities by allowing registered professionals to be reimbursed for performing tasks that non-registered practitioners cannot.

This principle does not mean that professions should cease to exist, rather that state recognition and monitoring should be based on the levels of competence and risk, rather than historic professional ‘bundles’ of skills. Instead, professions could continue to be supported through their respective professional bodies who could continue to uphold professional standards, such as quality, while reflecting patient-centred values.

Removing state protection of professional monopolies means that professions will have to compete with other providers to deliver those tasks. The professions will need to use other strategies to maintain their professional branding. There will be much greater self-interest by the professions to promote their brand and explicitly self-regulate on the basis of patient quality and safety. This is reinforced in the earlier example of the podiatric surgeons successfully competing with orthopaedic surgeons to deliver foot surgery by using explicit indicators of quality and efficiency.

Being a member of a professional body will be seen as an explicit endorsement of professional values, and professions will have greater self-interest in removing those members who fail to adhere to the standards defined by their profession.

One challenge of separating competencies from professions is a lack of consumer knowledge about *which* services to purchase, although there is little evidence to suggest that consumers or health-care purchasers are currently well informed to make judgements about the appropriateness or quality of health services outside of the hospital setting [68, 88, 89]. This may need to be addressed through brokerage, such as service commissioning on behalf of the service user. In many cases, this would simplify worker roles. For instance, aged care workers could be trained to in a wide range of skills necessary to treat older people (for example, foot care skills, support with eating, mobility), rather than engaging a multidisciplinary team who negotiate to perform these skills between them [90].

Regulation is an important component of this principle. The separation of regulation from professions provides a much more flexible and responsive way for new tasks or technologies to be introduced safely onto the market. The credentialing of practitioners based on individual competencies may sound onerous and time-consuming; however, this is the basis of most existing training courses for professions. Tools already exist to define and allocate tasks to appropriate practitioners on the basis of competence and risks [37].

The United Kingdom’s NHS introduced a competency-based framework, the Knowledge and Skills Framework (KSF) which standardised competency levels across all non-medical professionals; however, the ability of this model to enhance workforce flexibility is limited by the overlaying of traditional professional boundaries and career hierarchies [91]. The separation of ‘medical’ competencies from this framework also restricts the direct transfer of activity between a wide range of professionals, instead reinforcing the separation of medicine from non-medical professionals.

### Principle 4: Match rewards and indemnity to the levels of skill and risk required to perform a particular task, not professional title

Existing regulation and insurance systems are largely profession based. This principle builds on the concept of recognising competencies, rather than professions, and reimbursing levels of *skill and risk* as opposed to a traditional professional repertoire. It also draws on Atkinson’s concept of wage flexibility, in which payment is negotiated on the basis of the individual costs of employment, rather than collectively, by professional groups.

In other words, lower risk, less skilled tasks should attract a lower fee and therefore be performed by lower cost workers [92]. Such a model would rapidly fragment health work so that skills and risks were matched by the appropriate level of worker and reimbursement. Similarly, risks and indemnity levels would need to match specific tasks which would subsequently be reflected in pricing.

This type of competency-based model would also begin to challenge existing occupational monopolies. This point is well illustrated in the earlier example of the podiatric surgeons who successfully contested the delivery of foot surgery on the basis of quality and price. The role negotiations were facilitated by a managerial policies model during a time of orthopaedic workforce shortages. The result was the development of the micro-specialism of foot surgery performed by podiatrists, rather than orthopaedic surgeons, which is now integrated with mainstream surgical services in many jurisdictions in the United Kingdom. Other analyses of workforce redistribution have illustrated how managerial pressures in primary care lead to role devolution and evolution [93].

The use of diagnosis-related groups is one method of reimbursement that draws on a bundle of attributes surrounding a diagnosis or procedure that is not directly linked to professions, which have the ability to reward and influence health-care quality [94] and could lead to efficiencies through role redesign.

### Principle 5: Ensure that practitioners have all the skills they need to perform the tasks required to work in the environment in which they work

As outlined above, patient-centred care is often compromised because of therapeutic boundaries, where practitioners lack access to the full scope of technologies and techniques to be able to provide a full, therapeutic intervention.

Diabetes care is a good example. Australian guidelines for best practice in diabetes care [95] recommend input from a diabetes educator, suggesting ‘*The diabetes educator, within their scope of practice, can often spend more time than the general practitioner has available, consolidating the patient’s knowledge and skills regarding eating plan, physical activity, self-monitoring, medication usage, initiation and support with insulin therapy, foot care etc.*’ (p 19). The guidelines also recommend input from a range of other professionals, including podiatrists, dietitians, endocrinologist, exercise professionals and ophthalmologists, under the coordination of a GP. Diabetes affects around 1.7 million Australians, many of whom will have limited access to any of the guidelines’ suggested professionals. A truly patient-centred model would ensure high levels of accessibility to all of the *skills* necessary to treat and support people with diabetes, rather than having those skills devolved across a wide range of professional repertoires. Such a model can occur with multidisciplinary teams of practitioners skilled to deliver the specific tasks or, more efficiently, ensure that a single practitioner has the skills necessary to address the majority of patient needs. The task sharing approach developed by the rural allied health practitioners may be an appropriate model in this context.

However, this principle raises questions about the role of specialisation in the workforce. Specialisation involves the adoption of specific expertise, generally in a smaller proportion of the workforce, as there is less demand for these skills [7, 96]. To address this, there are several examples of ‘hub and spoke’ models of care, in which the more generalist practitioner delivers care closer to the patient with support from a more specialised practitioner who is located nearer more specialised facilities, for instance, the use of hub and spoke models of rural surgical support [97], the use of telehealth to deliver speech pathology [98] and the use of interdisciplinary support workers in community-based rehabilitation services whose roles incorporate tasks from range of different practitioners which they deliver directly to the patient with team-based supervision [99].

### Principle 6: Enable practitioners to work to their full scope of practice delegate tasks where required

An efficient workforce would have staff working to their full scope of practice the majority of the time and not performing tasks that can be delegated to other practitioners [100]. This principle relies on practitioners being able to delegate tasks that do not require their highest levels of expertise [8].

There is limited evidence suggesting that nurses and allied health practitioners do not work to their full scope of practice [101–103]. In many cases, the boundaries defining the scope of practice of a profession are unclear [102]. For most professions, the scope of practice is defined by competencies that are agreed by and endorsed by the professional body that oversees or regulates the workforce, adjusted to different contextual circumstances.

Several inefficiencies arise in health-care delivery because of barriers that prevent health practitioners from working to their full scope of practice [100, 104]. A recent Ministerial Taskforce commissioned by the Minister for Health in Queensland, Australia, identified numerous tasks in which allied health practitioners were educated, competent and authorised to practice, but restricted from performing these duties due to legislative, administrative, policy and traditional approaches to practice [43, 102]. Examples of these barriers included practitioners who normally have first contact status in the care pathway requiring patients to have referrals to access those services, diagnostic practitioners (such as radiographers) not being able to comment on investigations and practitioners unable to make direct referrals to other practitioners or services without going through an intermediary. The consequences of these therapeutic boundaries are additional clinical transactions for the patient, additional service costs and delays in health-care delivery. Queensland Health is introducing a suite of key performance indicators to remove the therapeutic partitions identified in the Ministerial Taskforce.

Disentangling roles from professions and the regulation and reimbursement for competencies, rather than professions, would enhance this approach.

## Conclusion

It makes little practical or economic sense for medically trained practitioners to insert or remove sutures or routinely give vaccinations or for a 4-year qualified podiatrist to cut toenails when a vocationally trained *micro-specialist* can deliver the role just as effectively [93, 105]. While these debates are not new [92], the barriers to workforce reform that rely on role renegotiation have been hampered by interdisciplinary role disputes still, largely under the banner of medical hegemony [93]. Negotiating a truly flexible workforce depends on being able to separate the politics from the roles and placing the patient at the centre of care delivery.

The 21st century health workforce needs to be able to respond to the changing demands of the population. The principles presented in this paper may seem contentious; however, they attempt to start to challenge the highly complex, social construction that reflects the way that health services are currently recognised and rewarded. Additionally, as the examples in this paper illustrate, many of these principles are already gradually being implemented, albeit, under different labels and on a small scale.

There is some evidence to suggest that the current workforce model is flawed, demonstrated by the widespread workforce re-engineering that takes place. For instance, the growth of interprofessionalism and transprofessionalism suggests market failure. Professions train in silos and are then retrained to work across boundaries, adopting aspects of another’s work relevant to the context. This model suggests that practitioners are not trained to be fit for purpose, resulting in inefficiencies and overtraining. Instead of reprofiling existing professions or workers, surely it makes sense to train practitioners appropriately for the context in which they will work.

The six principles presented in this paper propose that the 21st century workforce could be more flexibly and appropriately arranged around specific tasks that are organised in ways that meet the needs of the population, not the professions. Additionally, the revolving door between GPs, diagnostic, therapeutic and specialist services should be examined for the inefficiencies that could be removed if the gate-keeping functions and therapeutic partitions were removed.

The introduction of new workforce flexibilities has implications for service users, policy-makers, workforce planners, managers, service providers, third-party payers, regulators and educators. In particular, for the workforce to adopt new roles, new systems of regulation and accountability will be required to facilitate changes in the scope of practice, to ensure that the appropriate training and accreditation exist and to protect service users and workers.

A challenge of moving towards a portfolio of competencies is that current systems of professional reward and recognition are based largely on unidisciplinary specialisation [91]. Stepping off a traditional professional trajectory will require new models to support career pathways based on risk and competence, not historical professional hierarchies.

Educators will need to prepare the workforce to adapt to the changing requirements of modern health-care systems. The rapid growth of the unregulated workforce, such as support workers and allied health assistants, has quality and safety implications. Additionally, third-party payers and other funders will need to introduce mechanisms to recognise the roles and input of non-traditional providers of care.

Should the professions cease to exist and be replaced by a generic workforce? The principles proposed in this paper suggest a continuum, with a generic workforce at one end, defined by a contextually relevant bundle of tasks or competencies that can be repackaged according to the needs or requirements of the end user. At the other end of the continuum lie the professions, with professional branding, history, culture and reputation. The middle ground is currently negotiated through interprofessional and transprofessional role renegotiation, where professions largely preserve their own identity by retaining ‘core’, defining tasks and the renegotiation and reallocation of ‘peripheral’ tasks that are less essential to their core identity [39]. We are already seeing a growing generic workforce, particularly in the unregistered arena. However, generic workers continue to co-exist alongside the professions. Without large-scale policy and/or funding changes, it is likely that the generic workforce will continue to grow, while the professional exclusivity will gradually erode due to increasing competition for their core tasks [7]. The sociology literature suggests that professions are robust, self-preserving entities, particularly when they are supported by the state [106]. In the absence of regulatory support, it is possible that strong professional branding may provide a competitive edge by reinforcing the perceived value of the professions in the eyes of the public and policy-makers. However, in an increasingly de-regulated, competitive, consumer-led marketplace, the health workforce of the future is likely to look very different to the workforce of today.

Placing the service user, not the professions, at the centre of workforce models has the potential to radically reshape the way that health-care is delivered in the 21st century, while delivering efficiencies and new ways of working.

## Author’s information

SN is the Director of Research at the School of Health and Human Sciences, Southern Cross University, Australia.
